# Post-traumatic Stress Disorder in Family-witnessed Resuscitation of Emergency Department Patients

**DOI:** 10.5811/westjem.2020.6.46300

**Published:** 2020-08-24

**Authors:** Mert Erogul, Antonios Likourezos, Jodee Meddy, Victoria Terentiev, D’anna Davydkina, Ralph Monfort, Illya Pushkar, Thomas Vu, Madhu Achalla, Christian Fromm, John Marshall

**Affiliations:** Maimonides Medical Center, Department of Emergency Medicine, Brooklyn, New York

## Abstract

**Introduction:**

Family presence during emergency resuscitations is increasingly common, but the question remains whether the practice results in psychological harm to the witness. We examine whether family members who witness resuscitations have increased post-traumatic stress disorder (PTSD) symptoms at one month following the event.

**Methods:**

We identified family members of critically ill patients via our emergency department (ED) electronic health record. Patients were selected based on their geographic triage to an ED critical care room. Family members were called a median of one month post-event and administered the Impact of Event Scale-Revised (IES-R), a 22-item validated scale that measures post-traumatic distress symptoms and correlates closely with *Diagnostic and Statistical Manual of Mental Disorders-IV* criteria for post-traumatic stress disorder (PTSD). Family members were placed into two groups based on whether they stated they had witnessed the resuscitation (FWR group) or not witnessed the resuscitation (FNWR group). Data analyses included chi-square test, independent sample t-test, and linear regression controlling for gender and age.

**Results:**

A convenience sample of 423 family members responded to the phone interview: 250 FWR and 173 FNWR. The FWR group had significantly higher mean total IES-R scores: 30.4 vs 25.6 (95% confidence interval [CI], −8.73 to −0.75; P<.05). Additionally, the FWR group had significantly higher mean score for the subscales of avoidance (10.6 vs 8.1; 95% CI, −4.25 to −0.94; P<.005) and a trend toward higher score for the subscale of intrusion (13.0 vs 11.4; 95% CI, −3.38 to .028; P = .054). No statistical significant difference was noted between the groups in the subscale of hyperarousal (6.95 vs 6.02; 95% CI, −2.08 to 0.22; P=.121). All findings were consistent after controlling for age, gender, and immediate family member (spouse, parent, children, and grandchildren).

**Conclusion:**

Our results suggest that family members who witness ED resuscitations may be at increased risk of PTSD symptoms at one month. This is the first study that examines the effects of family visitation for an unsorted population of very sick patients who would typically be seen in the critical care section of a busy ED.

## INTRODUCTION

The issue of family-witnessed resuscitation (FWR) has been debated since the late eighties when doctors at Foote Hospital in Michigan published their observations suggesting a benefit to family members who stayed to watch their critically ill loved ones getting cardiopulmonary resuscitation (CPR).^1^ Since then, authors have considered the question from medico-legal and ethical perspectives, from practical standpoints related to physician stress and potential interference with resuscitation and from the angle of potential harm or benefit to the family members themselves.^2^–^7^ Even as this debate has continued, the practice has spread to many emergency departments (ED) in North America and worldwide. Numerous professional bodies have endorsed FWR.^7^–^12^

Despite the growing consensus, there remains the question of the remote psychological cost of this experience to the witness. In particular, concern has been raised about the development of post-traumatic stress disorder (PTSD) in witnesses of resuscitation.^13^–^16^ PTSD is a syndrome of hyperarousal, vivid re-living of events, and inability to achieve a state of safety that results from, among other things, exposure to the actual or threatened death of a family member or friend in a way that is “accidental or violent.”^17^,^18^ Symptoms must be present for a month and the pathophysiology is thought to relate to memory being encoded in alternative, more persistent pathways that resist being extinguished. The literature supports the development of PTSD immediately or up to months after witnessing traumatic events, but whether or not this happens is related to multiple factors including the victim’s interpretation of the event, their pre-existing beliefs, and prior experiences.^19^

ED resuscitations can be brutal and memorable events in which the human form is treated violently or disfigured by invasive procedures; so it stands to reason that they might constitute the kind of traumatic event that can trigger PTSD in the brain of the witness. The experimental literature about FWR and the development of PTSD is conflicting and not of high quality.^13^–^16^ Moreover, all of the studies thus far have limited themselves to the subset of patients getting CPR. There is no literature about the much broader category of critically patients who are resuscitated but do not receive CPR, yet family members frequently ask to be present for the presorted range of ED resuscitations, even if they do not involve CPR. In view of the noted gaps, the divergent outcomes, and uneven quality of the current literature, there is a clear justification for further study. Our objective was to assess whether family and close contacts who witness ED resuscitations exhibit an increase in PTSD symptoms in the months following an event.

## METHODS

### Study Design and Setting

We conducted a prospective cross-sectional study, from July 2011–June 2016, comparing family members who witnessed resuscitation (FWR group) and family members who did not witness resuscitation (FNWR group) to assess post-traumatic distress symptoms. We conducted this study at a 711-bed urban, community teaching hospital with an annual ED census of greater than 120,000 visits. The goal was to enroll 150 participants in each arm. Patient screening, enrollment, and data collection were performed by study investigators. The hospital’s institutional review board approved the study.

**Population Health Research Capsule**

What do we already know about this issue?

Family-witnessed resuscitation has become accepted in the emergency department (ED) despite the question of remote psychological cost of this experience to the witness.

What was the research question?

Do family who witness ED resuscitations exhibit an increase in PTSD symptoms in the months following an event?

What was the major finding of the study?

There was an association between family presence during resuscitation and increased PTSD symptoms at one month.

How does this improve population health?

Adding to the growing body of literature about family-witnessed resuscitation moves us closer to sound recommendations for family of critically ill ED patients.

### Selection of Participants

At our institution, critically ill patients who need immediate, lifesaving attention are treated in a specific resuscitation room. We generated a list of patients who were treated in the resuscitation room and identified family members of those patients via our ED electronic health record. We included family members of critically ill patients aged 18 and older who underwent resuscitation in the ED. We excluded non-English speaking patients and those whose primary resuscitations occurred out of hospital. We did not capture whether resuscitations were medical or related to trauma, or whether or not they were successful. Patients did not require CPR to be entered into the study, although many of them did get CPR, were intubated, or received other procedures in a time-sensitive manner. Family members were given the choice to be present in the room during all resuscitations and were called via telephone one month after the event. They were placed into two groups based on whether they self-reported that they had witnessed the resuscitation (FWR group) or had not witnessed the resuscitation (FNWR group).

### Data Collection

The phone interviewers, consisting of college-educated and trained research assistants, administered the Impact of Event Scale-Revised (IES-R), a reliable 22-item validated scale that measures post-traumatic distress symptoms and correlates closely with the *Diagnostic and Statistical Manual of Mental Disorders-IV* criteria for PTSD.^20^ It is best suited for recent, not remote, traumatic events. Interviewers made up to three attempts to contact study subjects between the hours of 8 am and 6 pm Monday through Friday. The total IES-R score ranges from 0 to 88, with scores >24 associated with clinical concern for PTSD or partial PTSD, >33 with probable PTSD, and >39 with PTSD severe enough to suppress immune system function, even 10 years after the impact.

The IES-R has three subscales that correspond to the classic features of PTSD, specifically the alternation between avoidance (deliberate efforts not to think about the event) and intrusion (nightmares, involuntary thoughts of the event, and interfering feelings). A third subscale assesses hyperarousal, which relates to persistence of sympathomimetic excitability such as feeling on guard, and experiencing sweats and palpitations. Subjects were asked how distressing each of 22 components of the IES-R was in the last week with respect to their family member’s illness event, getting from zero to four points depending on the severity of the distress. Composite scores above 24 suggest a clinical concern for PTSD, whereas scores above 37 are associated with symptoms profound enough to cause immune dysfunction.

### Outcomes Measures

The primary outcome included a difference in total IES-R score between the groups. Secondary outcomes included a comparative difference between the three subscale scores of intrusion, avoidance, and hyperarousal.

### Data Analyses

The investigators recorded all data on data sheets (separate from clinical data), entered them into Microsoft Excel (Microsoft Corporation, Redmond, WA), and then imported the data into SPSS 24.0 (IBM Corp, Armonk, NY) for statistical analyses. Data were described in terms of mean (standard deviation [SD]) or 95% confidence limits for continuous variables, and frequency (percentage) for categorical variables. Data analyses included chi-square test, independent sample t-test, and linear regression controlling for gender, age, and immediate family member (spouse, parent, children, and grandchildren). A P-value <.05 denoted statistical significance between the groups.

## RESULTS

An estimated 3000 family members qualified for the study, of which approximately 1200 were reached by the phone interviewers. A convenience sample of 423 family members completed the IES-R, a response rate of 35%. The median duration between traumatic event and interview was 33 days (range 18–67), and there was no difference between groups in this regard. Of the 423 family members who completed the survey, 250 self-reported in the FWR and 173 the FNWR group. Family members consisted of immediate family members (children, parents, spouses, or grandchildren) as well as close friends, cousins, nieces, nephews, aunts, daily caretakers, and in-laws. The FWR group had more immediate family members (82.7% vs. 73.4%; P<.05). The mean age for the FWR group was 53.5 (±14.7) and for the FNWR group was 53.4 (±15.1; P=.905). The FWR group consisted of 71.7% females and FNWR of 63.2% females (P=.069) (Table 1). The FWR group had significantly higher mean total IES-R scores: 30.4 vs 25.6 (95% confidence interval [CI], −8.73 to −0.75; P<.02). Additionally, the FWR group had a significantly higher mean score for the subscales of avoidance (10.6 v. 8.1; 95% CI: −4.25 to −0.94; P<.005) and a trend toward a higher score for the subscale of intrusion (13.0 vs 11.4; 95% CI: −3.38 to .028; P=.054). No statistically significant difference was found between the groups in the subscale of hyperarousal (6.95 v. 6.02; 95% CI, −2.08 to 0.22; P =.121). All findings were consistent after controlling for age, gender, and immediate family member (spouse, parent, children, and grandchildren) in a linear regression equation (Table 2).

## DISCUSSION

In our prospective cross-sectional study, we found an association between family presence during resuscitation and increased PTSD symptoms at one month as measured by the IES-R. The IES-R is reported as a composite score with three subscales. In our study, we found significant differences in the composite scores as well as in the avoidance subscale. There was also a trend toward significance for the intrusion subscale. Prior experimental studies as detailed below employed the IES-R as a measure of PTSD, although the IES-R does not capture hyperarousal symptoms, thus limiting our ability to compare and generalize with those studies.

Robinson et al^13^ conducted a small, prospective, semi-randomized survey of 13 family members who had witnessed ED resuscitation of cardiac arrest after being given the choice to do so, and 12 who were not given the choice to witness CPR (and who additionally did not ask to witness). Successful resuscitations were excluded. The family members were queried using the IES-R at one month and six months by mail. The study was stopped early because the participating staff became convinced of the value of providing family access to resuscitations. It was limited by the use of a multiplicity of outcome measures and found no significant difference between the two groups in the development of PTSD symptoms.

Compton et al^14^ conducted a small, prospective, non-randomized cohort study of 54 family members of patients who had undergone failed resuscitation of out-of-hospital arrest. The family members were surveyed using the PTSD Symptom Scale Interview (PSSI) by telephone a month after the ED visit. The 34 who had witnessed the CPR had considerably higher (almost double) PSSI measures than the 20 who had not. The study’s limitations included lack of randomization, blinding, participant decay, and differing characteristics between the study groups.

Compton et al 15 prospectively compared two hospitals, one at which families were permitted to witness non-traumatic resuscitations (#24) and another at which families were not (#41). The subjects were interviewed by telephone at one and two months and evaluated using the PTSD-self report and the Center for Epidemiologic Studies Depression scale tools. The only significant difference in bereavement-related PTSD symptoms between the groups was an increase in arousal in the FWR group at two months. There were pre-intervention differences between the two groups, possibly related to cachement populations of the respective hospitals. The study was small and not randomized.

The one randomized and controlled study to date was conducted by Jabre et al^16^ in Paris. In their health system, ambulances known as “mobile ICUs” [intensive care units] are staffed by emergency physicians. Half of the local ambulance teams gave family members the choice to be present and the other half did not. Ninety days after the resuscitation, a blinded psychologist conducted a telephone questionnaire that included the IES-R. They followed 570 family members for one year to compare psychological outcomes in those who had been given the option to be present during resuscitation (n = 239) and those who had not, as well as between those who had witnessed resuscitation and those who had not. The authors found that family members who had not witnessed resuscitation displayed significantly more PTSD symptoms than those who had (IES 26 to 21, p 0.007) (Table 3).

To summarize the findings of the above studies, one showed more PTSD symptoms with FWR, one showed fewer PTSD symptoms with FWR, and two showed no meaningful difference. Furthermore, there is a diversity of studied scenarios. Robinson excluded successful resuscitations from analysis. In another case, only cardiac arrest or violent trauma were included. Notably, all of these studies were limited to patients receiving CPR. Yet family members frequently ask to be present for all manner of ED resuscitations, even if they do not involve CPR. To our knowledge there is no data for family members who witness the type of presorted range of resuscitations that occur in the ED.

Our findings support the hypothesis that witnessing resuscitation, which is often sudden, unexpected, violent, or frightening to the observer, may lead to PTSD symptoms. This is the first study that examines the effect of family visitation in an unsorted population of very sick patients that would typically be seen in the critical care section of a busy ED. In contrast to the reviewed studies that excluded patients who survived, we did not track whether or not the resuscitations were successful. It can be argued that considering all-comers (successful resuscitation and not) is more relevant from a policy perspective as it is difficult to tell in advance whether or not a resuscitation will be effective and the important question is related to the strategy of how to manage family members of a presorted population of all critically ill patients. In studies limited to patients getting CPR – and particularly in those limited to patient deaths – family members may display a different pattern of symptoms intertwined in complicated ways with their grief reaction. The complex interrelationship between PTSD and bereavement in this context deserves further consideration.

In our study, the composite score and the avoidance subscore showed significant changes in the FWR group. The two other subscales did not show any changes. In the four prior cited studies, Compton (2008) suggested increased hyperarousal their FWR group and Compton (2011) showed an increase in hyperarousal at 60 days but not at 30 days. The other studies used the IES tool, which did not measure symptoms of hyperarousal.

Our study adds to the limited body of literature on the topic of PTSD symptoms in FWR in the ED. It is too soon to make evidence-grounded recommendations about whether or not families should be encouraged or permitted to witness resuscitations. For starters, the evidence is conflicting and incomplete – there is no consistent message in the literature. Moreover, a potential increase in PTSD symptoms is only one of many outcome measures that would be relevant to such a recommendation. In deciding whether to permit family members to witness resuscitations, one must weigh a slight increase in the development of PTSD symptoms as evidenced in our study against potential psychological benefits that are as yet unstudied. For instance, there is an emerging body of literature suggesting that PTSD symptoms such as rumination and intrusive thoughts may be a bridge to post-traumatic growth.^21^ Other measures such as development of complicated grief, acute stress disorder, and depression would also be relevant. Studies show that when asked, families overwhelmingly prefer to be given the option to be present.^22^–^25^ To limit autonomy based on paternalistic impulse is to defy the prevailing momentum of increased transparency in medicine set in motion during the era of patient rights fifty years ago, and should require a very high bar of evidence. At this point, there is still no reason to disagree with authors who advocate giving family members an informed choice to be present during resuscitations.^26^

## LIMITATIONS

The primary limitation of our design is that the sorting of subjects into FWR and FNWR groups was not randomized but rather left to the discretion of the participants. Subjects who chose to witness resuscitations may have done so because of closer bonds with their family member, and were therefore more likely to develop PTSD, potentially skewing the results. Additionally, the FWR group was somewhat more likely to include immediate family, although on regression analysis the presence of immediate family did not correlate with PTSD symptoms. There was a self-selection bias of the convenience sample of family members who agreed to participate. Finally, our analysis could have benefited from a reassessment of outcome measures at a later time point, as patients can develop PTSD symptoms even after many months.

## CONCLUSION

In our prospective cross-sectional study of an unsorted population of ED resuscitations, there was an association between family presence during resuscitation and increased PTSD symptoms as measured by the IES-R scale. There are numerous relevant questions that remain unanswered, and our suggestions for future research would include a longer follow-up period to assess later development of symptoms as well as inclusion of measures of post-traumatic psychological growth. There is also the question of whether the presence of social workers or chaplains or other informed guides might affect the outcome of witnessed resuscitations. Finally, it would be important to replicate this study in a pediatric patient population.

## Supplementary Information



## Figures and Tables

**Table 1:** Participant characteristics.

	FWR	FNWR
% Male	28.3	46.8
% Female	71.7	63.2
% Immediate family	82.7	73.4
Days from resuscitation to interview	33 (18–67)	33 (20–67)

*FWR*, family-witnessed resuscitation; *FNWR*, family not witnessed resuscitation.

**Table 2:** IRS-R Total mean scores and subscales intrusion, avoidance, and hyperarousal

	No FWR	FWR	Mean difference	95% CI	P-value
Mean total score	25.6	30.4	−4.74	−8.73 to −0.75	.020*
Mean intrusion score	11.4	13.0	−1.68	−3.38 to 0.028	.054
Mean avoidance	8.24	10.6	−2.33	−3.89 to −0.76	.003**
Mean hyperarousal	6.02	6.95	−0.93	−2.08 to 0.22	.121

* Statistical significant difference between the groups at P<.05;

** Statistical significant difference between the groups at P<.005.

*FWR*, family-witnessed resuscitation; *FNWR*, family not witnessed resuscitation; *CI*, confidence interval.

**Table 3:** Prior experimental studies of post-traumatic stress disorder in family-witnessed resuscitation.

Study	Inclusion criteria	PTSD outcome measures
Robinson, 1998^13^	ED cardiac arrest / multi-trauma	IES^1^
Compton, 2009^14^	Out of hospital cardiac arrest	PSSI^2^
Compton, 2011^15^	ED cardiac arrest	PSS-SR^3^
Jabre, 2014^16^	Out of hospital cardiac arrest	IES^1^

*PSSI*, Post-Traumatic Stress Disorder Symptom Scale Interview; *ED*, emergency department; *IES*, Impact of Events Scale; *PSS-SR*, post-traumatic stress disorder Symptom Scale—Self Report.
