# Supplementary material for: Post-traumatic Stress Disorder in Family-witnessed Resuscitation of Emergency Department Patients
Source: West J Emerg Med. 2020 Aug 24;21(5):1182–7. doi: 10.5811/westjem.2020.6.46300 (PMC7514396; doi:10.5811/westjem.2020.6.46300)
Supplement: Supplementary file 1 [file wjem-21-1182-s001.pdf]

## IMPACT OF EVENTS SCALE-Revised (IES-R)

INSTRUCTIONS: Below is a list of difficulties people sometimes have after stressful life events. Please read each item, and then indicate how distressing each difficulty has been for you DURING THE PAST SEVEN DAYS with respect to ____________________________ ____________________________________________________________(event) that occurred on ______________________(date). How much have you been distressed or bothered by these difficulties?

| | Not at all | A little bit | Moderately | Quite a bit | Extremely |
|---|---|---|---|---|---|
| 1. Any reminder brought back feelings about it | 0 | 1 | 2 | 3 | 4 |
| 2. I had trouble staying asleep | 0 | 1 | 2 | 3 | 4 |
| 3. Other things kept making me think about it. | 0 | 1 | 2 | 3 | 4 |
| 4. I felt irritable and angry | 0 | 1 | 2 | 3 | 4 |
| 5. I avoided letting myself get upset when I thought about it or was reminded of it | 0 | 1 | 2 | 3 | 4 |
| 6. I thought about it when I didn't mean to | 0 | 1 | 2 | 3 | 4 |
| 7. I felt as if it hadn't happened or wasn't real. | 0 | 1 | 2 | 3 | 4 |
| 8. I stayed away from reminders of it. | 0 | 1 | 2 | 3 | 4 |
| 9. Pictures about it popped into my mind. | 0 | 1 | 2 | 3 | 4 |
| 10. I was jumpy and easily startled. | 0 | 1 | 2 | 3 | 4 |
| 11. I tried not to think about it. | 0 | 1 | 2 | 3 | 4 |
| 12. I was aware that I still had a lot of feelings about it, but I didn't deal with them. | 0 | 1 | 2 | 3 | 4 |
| 13. My feelings about it were kind of numb. | 0 | 1 | 2 | 3 | 4 |
| 14. I found myself acting or feeling like I was back at that time. | 0 | 1 | 2 | 3 | 4 |
| 15. I had trouble falling asleep. | 0 | 1 | 2 | 3 | 4 |
| 16. I had waves of strong feelings about it. | 0 | 1 | 2 | 3 | 4 |
| 17. I tried to remove it from my memory. | 0 | 1 | 2 | 3 | 4 |
| 18. I had trouble concentrating. | 0 | 1 | 2 | 3 | 4 |
| 19. Reminders of it caused me to have physical reactions, such as sweating, trouble breathing, nausea, or a pounding heart. | 0 | 1 | 2 | 3 | 4 |
| 20. I had dreams about it. | 0 | 1 | 2 | 3 | 4 |
| 21. I felt watchful and on-guard. | 0 | 1 | 2 | 3 | 4 |
| 22. I tried not to talk about it. | 0 | 1 | 2 | 3 | 4 |

Total IES-R Score:________________

INT: 1, 2, 3, 6, 9, 14, 16, 20
AVD: 5, 7, 8, 11, 12, 13, 17, 22
HYP: 4, 10, 15, 18, 19, 21

Weiss, D.S. (2007). The Impact of Event Scale-Revised. In J.P. Wilson, & T.M. Keane (Eds.) *Assessing psychological trauma and PTSD: a practitioner's handbook* (2nd ed., pp. 168-189). New York: Guilford Press.

Revised Impact of Event Scale (22 questions):

The revised version of the Impact of Event Scale (IES-r) has seven additional questions and a scoring range of 0 to 88.

On this test, scores that exceed 24 can be quite meaningful. High scores have the following associations.

**Score** (IES-r) **Consequence**

| | |
|---|---|
| 24 or more | PTSD is a clinical concern.[6] Those with scores this high who do not have full PTSD will have partial PTSD or at least some of the symptoms. |
| 33 and above | This represents the best cutoff for a probable diagnosis of PTSD.[7] |
| 37 or more | **This is high enough to suppress your immune system's functioning** (even 10 years after an impact event).[8] |

The IES-R is very helpful in measuring the affect of routine life stress, everyday traumas and acute stress

**References:**


1. Horowitz, M. Wilner, N. & Alvarez, W. (1979). Impact of Event Scale: A measure of subjective stress. Psychosomatic Medicine, 41, 209-218.
2. Weiss, D.S., & Marmar, C.R. (1997). The Impact of Event Scale-Revised. In J.P. Wilson & T.M. Keane (Eds.), Assessing Psychological Trauma and PTSD (pp.399-411). New York: Guilford.
3. Hutchins, E. & Devilly, G.J. (2005). Impact of Events Scale. Victim's Web Site. http://www.swin.edu.au/victims/resources/assessment/ptsd/ies.html
4. Coffey, S.F. & Berglind, G. (2006). Screening for PTSD in motor vehicle accident survivors using PSS-SR and IES. Journal of Traumatic Stress. 19 (1): 119-128.
5. Neal, L.A., Walter, B., Rollins, J., et al. (1994). Convergent Validity of Measures of Post-Traumatic Stress Disorder in a Mixed Military and Civilian Population. Journal of Traumatic Stress. 7 (3): 447-455.
6. Asukai, N. Kato, H. et al. (2002). Reliability and validity of the Japanese-language version of the Impact of event scale-revised (IES-R-J). Journal of Nervous and Mental Disease. 190 (3): 175-182.
7. Creamer, M. Bell, R. & Falilla, S. (2002). Psychometric properties of the Impact of Event Scale-Revised. Behaviour Research and Therapy. 41: 1489-1496.
8. Kawamura, N. Yoshiharu, K. & Nozomu, A. (2001) Suppression of Cellular Immunity in Men with a Past History of Post Traumatic Stress Disorder. American Journal of Psychiatry. 158: 484-486
